# 3-Bromo­meth­yl-2-chloro­meth­yl-1-phenyl­sulfon­yl-1*H*-indole

**DOI:** 10.1107/S1600536808007678

**Published:** 2008-03-29

**Authors:** G. Chakkaravarthi, V. Dhayalan, A. K. Mohanakrishnan, V. Manivannan

**Affiliations:** a Department of Physics, CPCL Polytechnic College, Chennai 600 068, India; b Department of Organic Chemistry, University of Madras, Guindy Campus, Chennai 600 025, India; c Department of Physics, Presidency College, Chennai 600 005, India

## Abstract

In the title compound, C_16_H_13_BrClNO_2_S, the indole mean plane forms a dihedral angle of 73.59 (19)° with the phenyl ring. The mol­ecular structure is stabilized by weak intra­molecular C—H⋯O inter­actions. The Br atom is disordered over two positions with site occupancy factors of 0.7 and 0.3.

## Related literature

For related crystal structures, see: Chakkaravarthi *et al.* (2007 ▶, 2008 ▶). For the biological activities of indole derivatives, see: Chai *et al.* (2006 ▶); Nieto *et al.* (2005 ▶); Olgen & Coban (2003 ▶).
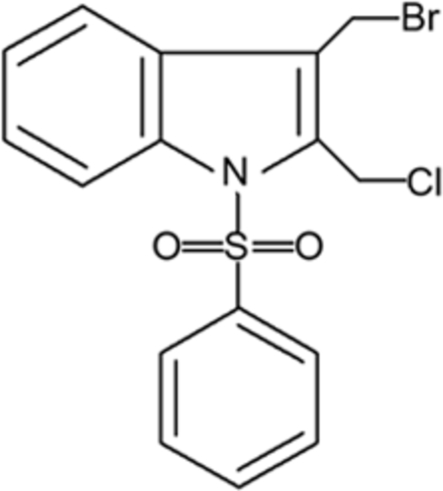


## Experimental

### 

#### Crystal data

C_16_H_13_BrClNO_2_S
*M* *_r_* = 398.69
Monoclinic, 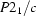

*a* = 11.8501 (9) Å
*b* = 16.3525 (13) Å
*c* = 8.5793 (6) Å
β = 108.766 (3)°
*V* = 1574.1 (2) Å^3^
*Z* = 4
Mo *K*α radiation
μ = 2.92 mm^−1^
*T* = 295 (2) K
0.16 × 0.14 × 0.14 mm

#### Data collection

Bruker Kappa APEXII diffractometer
Absorption correction: multi-scan (*SADABS*; Sheldrick, 1996 ▶) *T*_min_ = 0.559, *T*_max_ = 0.665
14367 measured reflections
2770 independent reflections
1822 reflections with *I* > 2σ(*I*)
*R*_int_ = 0.045

#### Refinement

*R*[*F* ^2^ > 2σ(*F* ^2^)] = 0.066
*wR*(*F* ^2^) = 0.231
*S* = 1.06
2770 reflections
209 parameters
12 restraints
H-atom parameters constrained
Δρ_max_ = 0.42 e Å^−3^
Δρ_min_ = −0.92 e Å^−3^

### 

Data collection: *APEX2* (Bruker, 2004 ▶); cell refinement: *APEX2*; data reduction: *APEX2*; program(s) used to solve structure: *SHELXS97* (Sheldrick, 2008 ▶); program(s) used to refine structure: *SHELXL97* (Sheldrick, 2008 ▶); molecular graphics: *PLATON* (Spek, 2003 ▶); software used to prepare material for publication: *SHELXL97*.

## Supplementary Material

Crystal structure: contains datablocks I, global. DOI: 10.1107/S1600536808007678/bt2689sup1.cif

Structure factors: contains datablocks I. DOI: 10.1107/S1600536808007678/bt2689Isup2.hkl

Additional supplementary materials:  crystallographic information; 3D view; checkCIF report

## Figures and Tables

**Table 1:** Hydrogen-bond geometry (Å, °)

*D*—H⋯*A*	*D*—H	H⋯*A*	*D*⋯*A*	*D*—H⋯*A*
C6—H6⋯O2	0.93	2.51	2.877 (9)	104
C13—H13⋯O1	0.93	2.31	2.873 (10)	118
C15—H15*B*⋯O2	0.97	2.17	2.939 (10)	136

**Fig. 1.**
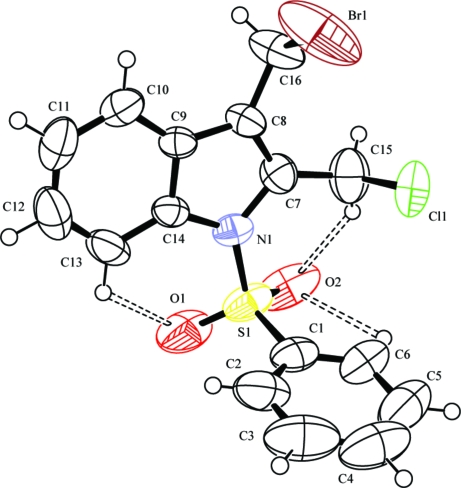
The molecular structure of (I) showing the atomic labels and 50% probability displacement ellipsoids for non-H atoms. Only major parts of the disordered atoms are drawn. Intramolecular H-bonds are shown as dashed lines.

## supplementary crystallographic information

### Comment

In continuation of our studies of indole derivatives, which are known to exhibit anti-oxidant activity (Olgen & Coban, 2003), antihepatitis B virus activities (Chai *et al.*, 2006) and antibacterial (Nieto *et al.*, 2005) activities, we report the crystal structure of the title compound (I).

The geometric parameters of the molecule of (I) (Fig. 1) agree well with the reported structures (Chakkaravarthi *et al.*, 2007, 2008). The indole mean plane forms a dihedral angle of 73.59 (19)° with the phenyl ring. The N1—S1—C1 plane is almost orthogonal to indole ring (dihedral angle 82.30 (22)°) and makes 76.93 (22)° with the phenyl ring. The indole mean plane and C8—C16—BR1 plane are nearly orthogonal to each other forming a dihedral angle of 82.23 (0.29)°.

The sum of bond angles around N1 (359.99°) shows that N1 is *sp*^2^-hybridized. The torsion angles O1—S1—N1—C14 and O2—S1—N1—C7 [17.8 (6)° and -33.4 (6)°, respectively] indicate the *syn* conformation of the sulfonyl moiety. The molecular structure is stabilized by weak intramolecular C—H···O interactions.

### Experimental

1-(Phenylsulfonyl)-3-(bromomethyl)-2-methylindole (0.5 g, 1.37 mmol) was dissolved in dry ccl_4_(10 ml) and then powdered *N*-chloro succinimide was added. To this, azobisisobutyronitrle (50 mg) was also added and then refluxed for 2 h on a waterbath. After the reaction was completed, succinimide was floated on the surface of the reaction mixture. It was then filtered off and washed with CCl_4_ (3 ml). The solvent was removed carefully under vacuo. The crude product was recrystallized from CCl_4_. Yield:76 percentage.

### Refinement

H atoms were positioned geometrically and refined using riding model with C—H = 0.93 Å and *U*_iso_(H) = 1.2Ueq(C) for aromatic C—H and C—H = 0.97 Å and *U*_iso_(H) = 1.2Ueq(C) for CH_2_. The Br atom is disordered over two positions with the occupancies of 0.709 (16) and 0.291 (16), respectively. The distances C1—C2, C2—C3, C3—C4, C4—C5, C10—C11 and C11—C12 were restrained to 1.395 (1) Å, the distances C16—BR1 and C16—Br1A were restrained to 1.91 (10) Å and the distance CL1—C15 was restrained to 1.76 (5) Å. The anisotropic thermal parameters of C15, C16, BR1, BR1A, CL1 atoms were restrained with DELU in the final cycles of the refinement (Sheldrick, 2008).

### Crystal data

**Table:**

C_16_H_13_BrClNO_2_S	*F*_000_ = 800
*M**_r_* = 398.69	*D*_x_ = 1.682 Mg m^−^^3^
Monoclinic, *P*2_1_/*c*	Mo *K*α radiation λ = 0.71073 Å
Hall symbol: -P 2ybc	Cell parameters from 4037 reflections
*a* = 11.8501 (9) Å	θ = 2.5–25.0º
*b* = 16.3525 (13) Å	µ = 2.92 mm^−^^1^
*c* = 8.5793 (6) Å	*T* = 295 (2) K
β = 108.766 (3)º	Block, colourless
*V* = 1574.1 (2) Å^3^	0.16 × 0.14 × 0.14 mm
*Z* = 4	

### Data collection

**Table:**

Bruker Kappa APEXII diffractometer	2770 independent reflections
Radiation source: fine-focus sealed tube	1822 reflections with *I* > 2σ(*I*)
Monochromator: graphite	*R*_int_ = 0.045
*T* = 295(2) K	θ_max_ = 25.0º
ω and φ scans	θ_min_ = 2.2º
Absorption correction: multi-scan(SADABS; Sheldrick, 1996)	*h* = −14→13
*T*_min_ = 0.559, *T*_max_ = 0.665	*k* = −19→19
14367 measured reflections	*l* = −10→10

### Refinement

**Table:**

Refinement on *F*^2^	Secondary atom site location: difference Fourier map
Least-squares matrix: full	Hydrogen site location: inferred from neighbouring sites
*R*[*F*^2^ > 2σ(*F*^2^)] = 0.066	H-atom parameters constrained
*wR*(*F*^2^) = 0.231	*w* = 1/[σ^2^(*F*_o_^2^) + (0.127*P*)^2^ + 1.9456*P*] where *P* = (*F*_o_^2^ + 2*F*_c_^2^)/3
*S* = 1.06	(Δ/σ)_max_ < 0.001
2770 reflections	Δρ_max_ = 0.42 e Å^−^^3^
209 parameters	Δρ_min_ = −0.91 e Å^−^^3^
12 restraints	Extinction correction: none
Primary atom site location: structure-invariant direct methods	

### Fractional atomic coordinates and isotropic or equivalent isotropic displacement parameters (Å^2^)

**Table:**

	*x*	*y*	*z*	*U*_iso_*/*U*_eq_	Occ. (<1)
Cl1	0.90925 (16)	−0.10531 (12)	0.5085 (3)	0.0872 (6)	
Br1	0.8135 (6)	0.1043 (5)	0.3205 (5)	0.1572 (16)	0.709 (16)
Br1A	0.7901 (4)	0.1251 (3)	0.3139 (9)	0.093 (2)	0.291 (16)
S1	0.70049 (14)	−0.13649 (11)	0.8192 (2)	0.0743 (6)	
O1	0.6340 (5)	−0.1191 (4)	0.9258 (8)	0.114 (2)	
O2	0.6743 (5)	−0.2077 (3)	0.7212 (8)	0.109 (2)	
N1	0.6795 (4)	−0.0573 (3)	0.6922 (5)	0.0539 (11)	
C1	0.8512 (6)	−0.1369 (4)	0.9320 (7)	0.0655 (17)	
C2	0.8952 (7)	−0.0826 (5)	1.0604 (8)	0.097 (2)	
H2	0.8449	−0.0449	1.0854	0.117*	
C3	1.0161 (8)	−0.0849 (8)	1.1523 (11)	0.127 (4)	
H3	1.0474	−0.0485	1.2387	0.153*	
C4	1.0885 (9)	−0.1418 (7)	1.1133 (13)	0.122 (4)	
H4	1.1688	−0.1441	1.1753	0.147*	
C5	1.0442 (8)	−0.1951 (6)	0.9845 (13)	0.113 (3)	
H5	1.0947	−0.2325	0.9590	0.135*	
C6	0.9256 (7)	−0.1932 (5)	0.8934 (10)	0.086 (2)	
H6	0.8953	−0.2295	0.8065	0.103*	
C7	0.7000 (5)	−0.0556 (3)	0.5384 (7)	0.0574 (14)	
C8	0.6681 (5)	0.0197 (3)	0.4715 (6)	0.0535 (14)	
C9	0.6288 (5)	0.0680 (3)	0.5809 (5)	0.0463 (12)	
C10	0.5887 (6)	0.1477 (4)	0.5707 (7)	0.0622 (15)	
H10	0.5837	0.1796	0.4790	0.075*	
C11	0.5563 (6)	0.1790 (4)	0.6984 (8)	0.078 (2)	
H11	0.5295	0.2327	0.6939	0.094*	
C12	0.5633 (7)	0.1313 (5)	0.8332 (9)	0.083 (2)	
H12	0.5406	0.1537	0.9182	0.099*	
C13	0.6026 (6)	0.0519 (5)	0.8471 (6)	0.0687 (18)	
H13	0.6064	0.0203	0.9388	0.082*	
C14	0.6365 (4)	0.0206 (3)	0.7184 (6)	0.0468 (12)	
C15	0.7557 (4)	−0.1232 (4)	0.4743 (11)	0.086 (2)	
H15A	0.7151	−0.1294	0.3572	0.103*	
H15B	0.7464	−0.1739	0.5278	0.103*	
C16	0.6722 (5)	0.0443 (4)	0.3064 (7)	0.084 (2)	
H16A	0.6030	0.0777	0.2520	0.101*	0.709 (16)
H16B	0.6686	−0.0042	0.2400	0.101*	0.709 (16)
H16C	0.6052	0.0802	0.2560	0.101*	0.291 (16)
H16D	0.6645	−0.0033	0.2363	0.101*	0.291 (16)

### Atomic displacement parameters (Å^2^)

**Table:**

	*U*^11^	*U*^22^	*U*^33^	*U*^12^	*U*^13^	*U*^23^
Cl1	0.0663 (11)	0.0872 (13)	0.1176 (15)	0.0181 (9)	0.0429 (10)	−0.0109 (10)
Br1	0.235 (3)	0.159 (3)	0.106 (2)	−0.112 (2)	0.095 (2)	−0.0245 (15)
Br1A	0.063 (4)	0.105 (3)	0.098 (3)	−0.014 (2)	0.008 (2)	0.041 (3)
S1	0.0540 (9)	0.0688 (11)	0.0926 (12)	−0.0071 (8)	0.0129 (8)	0.0402 (9)
O1	0.082 (3)	0.142 (5)	0.130 (5)	0.015 (3)	0.053 (3)	0.087 (4)
O2	0.087 (3)	0.059 (3)	0.144 (5)	−0.029 (3)	−0.016 (3)	0.035 (3)
N1	0.057 (3)	0.049 (3)	0.052 (2)	0.000 (2)	0.012 (2)	0.013 (2)
C1	0.061 (4)	0.067 (4)	0.065 (4)	−0.010 (3)	0.015 (3)	0.028 (3)
C2	0.100 (6)	0.123 (7)	0.058 (4)	−0.002 (5)	0.010 (4)	0.011 (4)
C3	0.123 (8)	0.145 (10)	0.079 (6)	−0.039 (8)	−0.016 (6)	0.010 (6)
C4	0.077 (6)	0.138 (9)	0.121 (8)	−0.020 (6)	−0.010 (6)	0.051 (7)
C5	0.076 (6)	0.116 (7)	0.139 (8)	0.024 (5)	0.025 (6)	0.041 (7)
C6	0.072 (5)	0.077 (5)	0.096 (5)	0.011 (4)	0.009 (4)	0.019 (4)
C7	0.064 (4)	0.051 (3)	0.059 (3)	−0.005 (3)	0.023 (3)	−0.008 (3)
C8	0.070 (4)	0.053 (3)	0.039 (3)	−0.007 (3)	0.020 (2)	−0.002 (2)
C9	0.047 (3)	0.052 (3)	0.034 (2)	−0.002 (2)	0.005 (2)	−0.001 (2)
C10	0.066 (4)	0.050 (3)	0.061 (3)	0.006 (3)	0.007 (3)	0.005 (3)
C11	0.066 (4)	0.066 (4)	0.095 (5)	0.014 (3)	0.014 (4)	−0.021 (4)
C12	0.071 (4)	0.106 (6)	0.073 (4)	0.003 (4)	0.028 (4)	−0.036 (4)
C13	0.062 (4)	0.104 (5)	0.037 (3)	−0.001 (4)	0.013 (3)	−0.002 (3)
C14	0.043 (3)	0.057 (3)	0.038 (2)	−0.003 (2)	0.009 (2)	0.004 (2)
C15	0.079 (3)	0.074 (5)	0.110 (6)	−0.007 (4)	0.039 (4)	−0.026 (4)
C16	0.134 (5)	0.073 (4)	0.051 (3)	−0.026 (4)	0.038 (4)	0.001 (3)

### Geometric parameters (Å, °)

**Table:**

Cl1—C15	1.772 (4)	C7—C8	1.359 (8)
Br1—C16	1.9114 (11)	C7—C15	1.481 (9)
Br1A—C16	1.9084 (11)	C8—C9	1.415 (8)
S1—O2	1.411 (6)	C8—C16	1.489 (7)
S1—O1	1.415 (6)	C9—C10	1.381 (8)
S1—N1	1.659 (4)	C9—C14	1.390 (7)
S1—C1	1.737 (6)	C10—C11	1.371 (7)
N1—C14	1.417 (7)	C10—H10	0.9300
N1—C7	1.417 (7)	C11—C12	1.375 (8)
C1—C2	1.380 (8)	C11—H11	0.9300
C1—C6	1.386 (10)	C12—C13	1.372 (10)
C2—C3	1.396 (8)	C12—H12	0.9300
C2—H2	0.9300	C13—C14	1.387 (8)
C3—C4	1.379 (9)	C13—H13	0.9300
C3—H3	0.9300	C15—H15A	0.9700
C4—C5	1.371 (9)	C15—H15B	0.9700
C4—H4	0.9300	C16—H16A	0.9700
C5—C6	1.371 (12)	C16—H16B	0.9700
C5—H5	0.9300	C16—H16C	0.9700
C6—H6	0.9300	C16—H16D	0.9700
			
O2—S1—O1	119.3 (4)	C9—C10—H10	120.6
O2—S1—N1	107.2 (3)	C10—C11—C12	120.3 (6)
O1—S1—N1	105.7 (3)	C10—C11—H11	119.9
O2—S1—C1	108.5 (3)	C12—C11—H11	119.9
O1—S1—C1	109.1 (4)	C13—C12—C11	122.5 (6)
N1—S1—C1	106.2 (3)	C13—C12—H12	118.8
C14—N1—C7	108.0 (4)	C11—C12—H12	118.8
C14—N1—S1	125.7 (4)	C12—C13—C14	117.1 (6)
C7—N1—S1	126.3 (4)	C12—C13—H13	121.4
C2—C1—C6	120.8 (7)	C14—C13—H13	121.4
C2—C1—S1	119.9 (6)	C13—C14—C9	120.9 (5)
C6—C1—S1	119.3 (5)	C13—C14—N1	131.9 (5)
C1—C2—C3	119.3 (8)	C9—C14—N1	107.2 (4)
C1—C2—H2	120.4	C7—C15—Cl1	111.8 (4)
C3—C2—H2	120.4	C7—C15—H15A	109.3
C4—C3—C2	119.1 (9)	Cl1—C15—H15A	109.3
C4—C3—H3	120.4	C7—C15—H15B	109.3
C2—C3—H3	120.4	Cl1—C15—H15B	109.3
C5—C4—C3	121.2 (9)	H15A—C15—H15B	107.9
C5—C4—H4	119.4	C8—C16—Br1A	113.8 (4)
C3—C4—H4	119.4	C8—C16—Br1	112.0 (4)
C6—C5—C4	120.1 (9)	C8—C16—H16A	109.2
C6—C5—H5	119.9	Br1A—C16—H16A	97.1
C4—C5—H5	119.9	Br1—C16—H16A	109.2
C5—C6—C1	119.5 (8)	C8—C16—H16B	109.2
C5—C6—H6	120.3	Br1A—C16—H16B	118.6
C1—C6—H6	120.3	Br1—C16—H16B	109.2
C8—C7—N1	107.6 (5)	H16A—C16—H16B	107.9
C8—C7—C15	128.1 (6)	C8—C16—H16C	108.4
N1—C7—C15	124.2 (6)	Br1A—C16—H16C	94.7
C7—C8—C9	109.4 (5)	Br1—C16—H16C	107.0
C7—C8—C16	123.8 (6)	H16B—C16—H16C	111.0
C9—C8—C16	126.7 (5)	C8—C16—H16D	110.3
C10—C9—C14	120.5 (5)	Br1A—C16—H16D	119.9
C10—C9—C8	131.7 (5)	Br1—C16—H16D	110.9
C14—C9—C8	107.8 (5)	H16A—C16—H16D	104.9
C11—C10—C9	118.7 (5)	H16C—C16—H16D	108.0
C11—C10—H10	120.6		
			
O2—S1—N1—C14	146.0 (5)	C15—C7—C8—C16	7.1 (9)
O1—S1—N1—C14	17.8 (6)	C7—C8—C9—C10	179.3 (6)
C1—S1—N1—C14	−98.1 (5)	C16—C8—C9—C10	−2.1 (10)
O2—S1—N1—C7	−33.4 (6)	C7—C8—C9—C14	−0.3 (6)
O1—S1—N1—C7	−161.6 (5)	C16—C8—C9—C14	178.3 (5)
C1—S1—N1—C7	82.5 (5)	C14—C9—C10—C11	−0.2 (8)
O2—S1—C1—C2	−167.5 (6)	C8—C9—C10—C11	−179.8 (6)
O1—S1—C1—C2	−36.0 (6)	C9—C10—C11—C12	−0.4 (10)
N1—S1—C1—C2	77.6 (6)	C10—C11—C12—C13	0.3 (11)
O2—S1—C1—C6	11.3 (6)	C11—C12—C13—C14	0.4 (10)
O1—S1—C1—C6	142.8 (5)	C12—C13—C14—C9	−0.9 (8)
N1—S1—C1—C6	−103.7 (5)	C12—C13—C14—N1	−179.4 (6)
C6—C1—C2—C3	−0.2 (11)	C10—C9—C14—C13	0.9 (8)
S1—C1—C2—C3	178.6 (7)	C8—C9—C14—C13	−179.4 (5)
C1—C2—C3—C4	−0.5 (15)	C10—C9—C14—N1	179.7 (5)
C2—C3—C4—C5	1.1 (17)	C8—C9—C14—N1	−0.6 (6)
C3—C4—C5—C6	−1.0 (16)	C7—N1—C14—C13	179.9 (6)
C4—C5—C6—C1	0.3 (13)	S1—N1—C14—C13	0.4 (8)
C2—C1—C6—C5	0.2 (11)	C7—N1—C14—C9	1.3 (6)
S1—C1—C6—C5	−178.5 (6)	S1—N1—C14—C9	−178.2 (4)
C14—N1—C7—C8	−1.5 (6)	C8—C7—C15—Cl1	76.0 (8)
S1—N1—C7—C8	178.0 (4)	N1—C7—C15—Cl1	−98.7 (7)
C14—N1—C7—C15	174.1 (5)	C7—C8—C16—Br1A	−112.1 (6)
S1—N1—C7—C15	−6.4 (8)	C9—C8—C16—Br1A	69.5 (8)
N1—C7—C8—C9	1.1 (6)	C7—C8—C16—Br1	−98.2 (7)
C15—C7—C8—C9	−174.3 (5)	C9—C8—C16—Br1	83.4 (8)
N1—C7—C8—C16	−177.5 (5)		

### Hydrogen-bond geometry (Å, °)

**Table:**

*D*—H···*A*	*D*—H	H···*A*	*D*···*A*	*D*—H···*A*
C6—H6···O2	0.93	2.51	2.877 (9)	104
C13—H13···O1	0.93	2.31	2.873 (10)	118
C15—H15B···O2	0.97	2.17	2.939 (10)	136
